# An India soyabean dataset for identification and classification of diseases using computer-vision algorithms

**DOI:** 10.1016/j.dib.2024.110216

**Published:** 2024-02-22

**Authors:** Jameer Kotwal, Ramgopal Kashyap, Mohd. Shafi Pathan

**Affiliations:** a Amity University Chhattisgarh, 493225, India; b MITSOC, MIT ADT University, 412201, India

**Keywords:** Soyabean leaf (Glycine max), Datasets, Image classification, Machine learning, Deep learning

## Abstract

Intelligent agriculture heavily relies on the science of agricultural disease image recognition. India is also responsible for large production of French beans, accounting for 37.25% of total production. In India from south region of Maharashtra state this crop is cultivated thrice in year. Soyabean plant is planted between the months of June through July, during the months of October and September during the rabi season, as well as in February. In the Maharashtrian regions of Pune, Satara, Ahmednagar, Solapur, and Nashik, among others, Soyabean plant is a common crop. In Maharashtra, Soyabean plant is grown over an area of around 31,050 hectares. This research presents a dataset of leaves from soyabean plants that are both insect-damaged and healthy. Images were taken over the course of fewer than two to three seasons on several farms. There are 3363 photos altogether in the seven folders that make up the dataset. Six categories comprise the dataset: I) Healthy plants II) Vein Necrosis III) Dry leaf IV) Septoria brown spot V) Root images VI) Bacterial leaf blight. This study's goal is to give academics and students accessibility to our dataset so they may use it for their studies and to build machine learning models.

Specifications Table

**Table:** 

Subject	Agronomy, Agricultural science, Horticulture, Computer science
Specific subject area	Image Processing, Deep Learning, plant diseases
Data format	Raw and filtered images are in .jpg format.
Type of data	Raw and preprocess images of Soyabean leaf
Data collection	Two smartphones are used to manually take high-quality photos. photos taken on a beautiful day. The top of the leaf is more heavily scrutinised to determine if it is healthy or unhealthy.
Data source location	Goudgaon Village farm of (Sub. Major Gulab Alam Kotwal), Tal: Barshi, Dist: Solapur, Maharashtra, India.413406. 18.2157727 Latitude and 75.6680118 Longitude.
Data accessibility	Repository name: An India soyabean leaf dataset Data identification number: 10.17632/bshkvgbzpt.1 Direct URL to data: https://data.mendeley.com/datasets/bshkvgbzpt/1 Instructions for accessing these data: Datasets consist of Single leaf and multi-leaf folder.
Related research article	**Case study:** Author: Mr.Jameer Kotwal, Dr.Ramgopal Kashyap, Dr.Shafi Pathan Paper: https://link.springer.com/article/10.1007/s11042-023-16882 Journal: Multimedia Tools and Application [1].

## 1 Value of the Data

• The dataset presented here is a collection of leaves from Soyabean plant that were gathered using mobile devices.
• Researchers as well as learners from many fields can use the dataset, which comprises of 1500 processed photos [2]. Researchers may utilise the dataset to review and validate the data as needed using various Predictive model, and to evaluate the precision of the algorithms.
• The dataset is a freely downloadable open source that is accessible to the general audience. So, without performing any additional pre-processing or confirmation, researchers may train the machine learning model using this dataset.
• The information may be used to develop high-quality tools for identifying and categorising diseases in Soyabean plant leaves that benefit society [3].

## 2 Background

### 2.1 Objectives

(a) A dataset with several disease classifications present on Soyabean plant leaves can aid AI/ML algorithms in real-time illness detection and classification.
(b) Pre-processing a dataset can help an AI/ML model perform more accurately.

## 3 Data Description

The six classifications that make up this dataset are healthy, vein necrosis, dry leaf, Septoria brown spot, root images and bacteria leaf blight. The first folder has 288 healthy images (Single leaf image and multi-image leaf). The second folder has 138 images of vein necrosis (Single leaf image and multi-image leaf). The dry leaf is in the third folder with 230 images (Single leaf image and multi-image leaf). Fourth folder contains 284 images of Septoria brown spot. The fifth folder contain 10 images of root. The sixth folder 226 images of bacteria leaf blight, while leaf images all(raw) is the last folder with 2187 images (Table 1).

**Table 1:** Types of disease and number of images.

Folder	Number of images
Healthy	288
Vein necrosis	138
Dry leaf	230
Septoria brown spot	284
Root images	10
Bacteria leaf blight	226

**Fig. 1:**
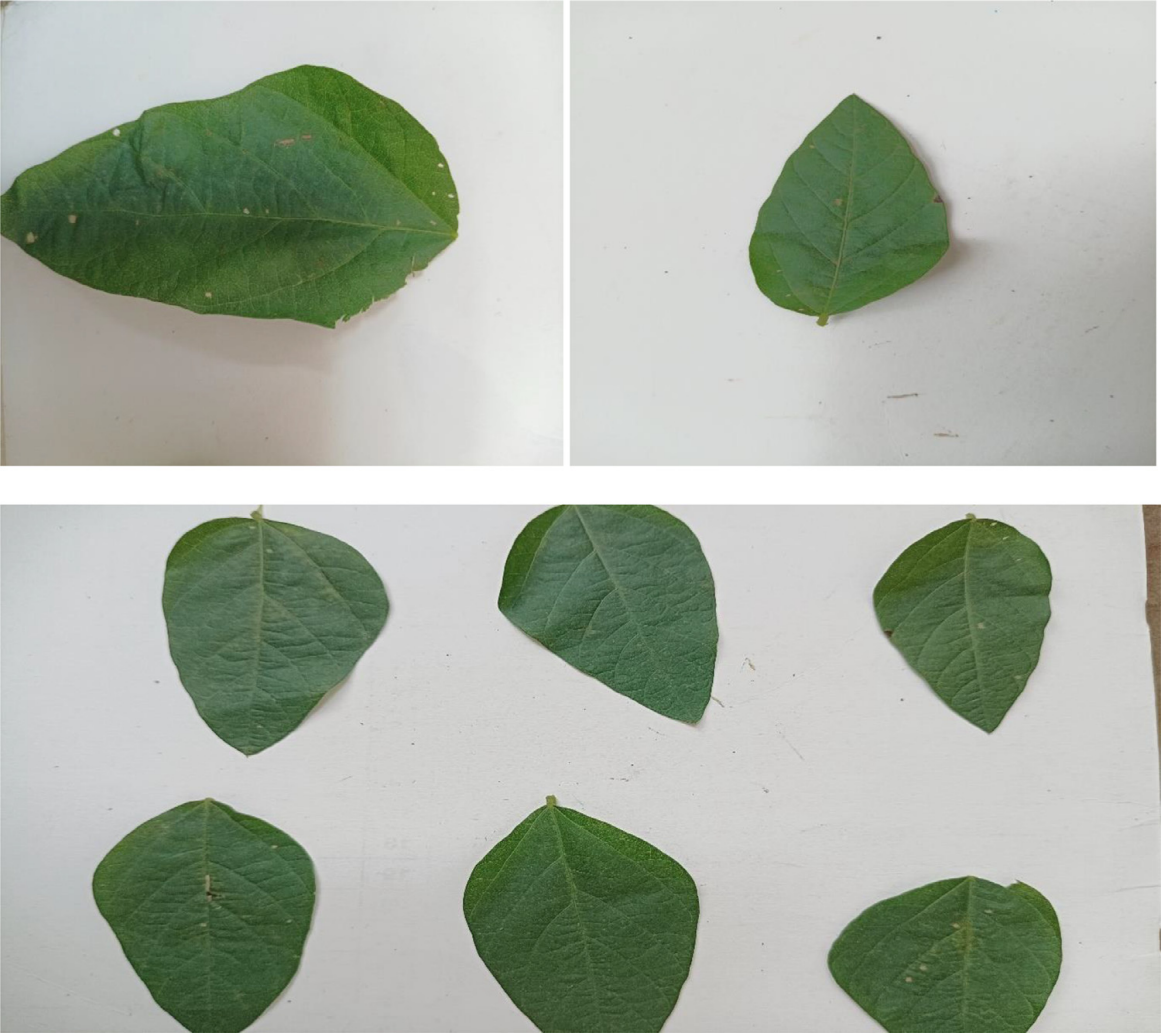
Healthy leaf with single and multi-image.

In this part, we examine the unusual symptoms of several diseases identified in our dataset's leaf photos. Examples of each ailment and the healthy group are shown in Fig. 1.

Vein necrosis is brought on by a fungus that needs water on the surface of leaves to flourish, thus watering at the plant's base will help eliminate moisture on the leaves.

One of the most destructive diseases of the common bean in tropical and subtropical production zones is Bacterial leaf spot (BLS), which is brought on by the bacterium pheudocercospora griseola.

**Fig. 2:**
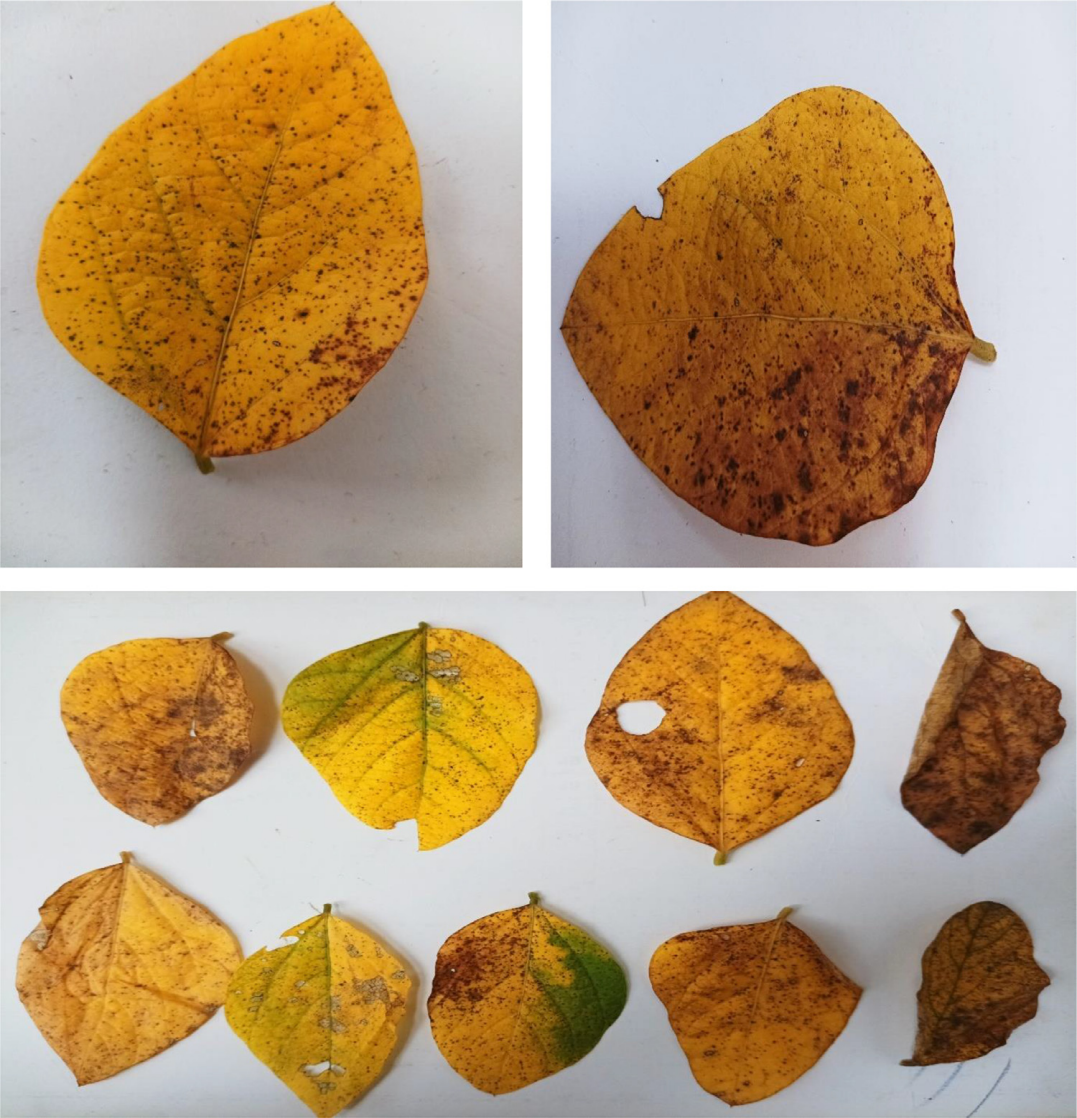
Single leaf and Multi-leaf of Septoria brown disease.

Common Septoria brown spot illness fusarium wilt has symptoms that resemble verticillium wilt. Yellowing, stunting, and deadness of seedlings are among the symptoms, as are yellowing and stunting of older plants (Fig. 2).

## 4 Experimental Design, Materials and Methods

Images from smartphones were taken in July 2022 from a small village called Goudgaon, Tal: Barshi, Dist: Solapur, in the Maharashtra area. Since the timeframe is ideal for Soyabean plant in the area, the procedure of taking the pictures took place at that time. Plants are photographed under sunny conditions at various stages.

Three stages made up the pre-processing of the photos.

**Fig. 3:**
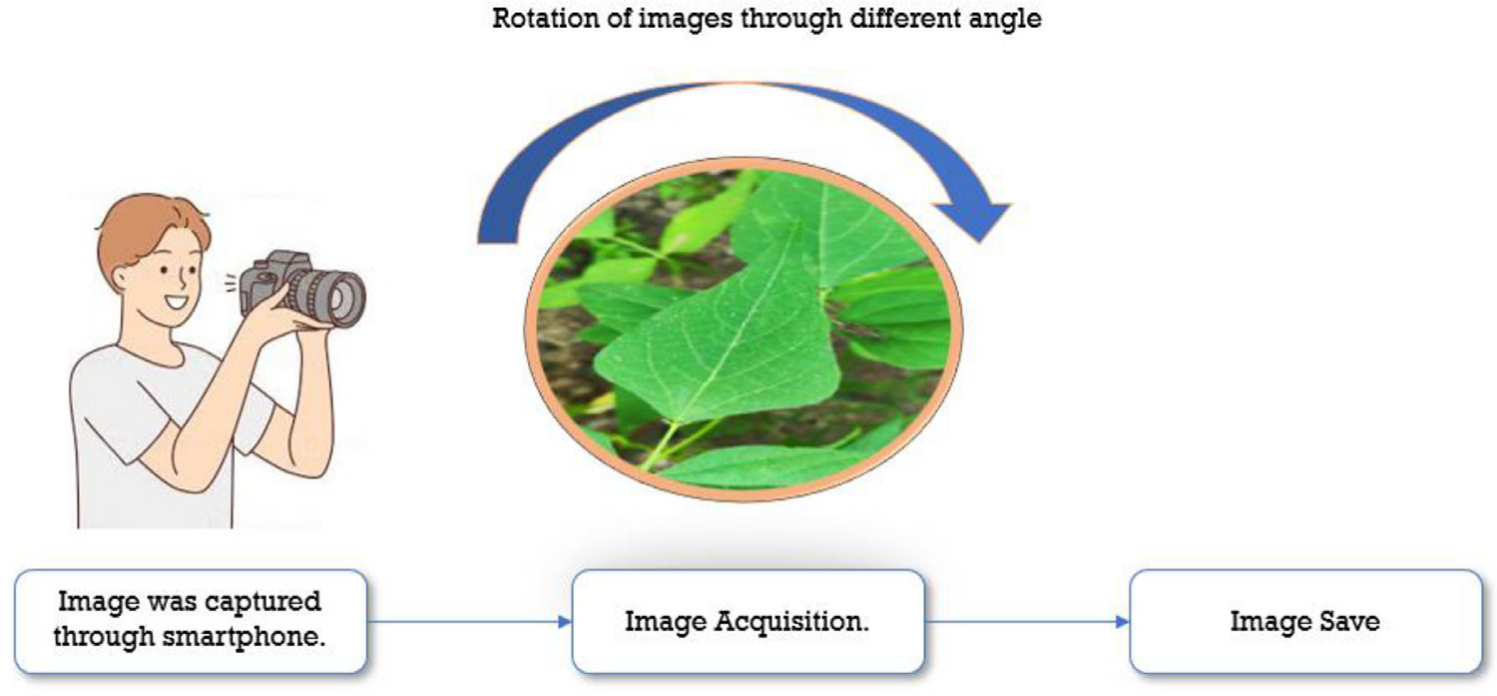
Collection of dataset.

(1) Data Acquisition:

The photographs were taken using the high-quality back camera of a smart phone. 3363 pictures were all taken using a camera, sorted, and stored to the appropriate folder as shown in Fig. 3.

(2) Image size:

In this phase, the images of different size collected from a village goudgaon situated in Maharashtra, India.

(3) Dataset split:

Separating a dataset into test and training sets to assess how well a machine learning model works. Splitting is necessary to solve the issue of overfitting.

The first phases of pre-processing are to arrange the images into six folders for the classification purpose: 1) Healthy 2) Vein necrosis 3) Dry leaf 4) Septoria brown spot 5) Root images 6) Bacteria leaf blight.

The second step is one of the most crucial since photographs are taken using smartphones, varying in size from 1600 × 1200 pixels in width and height to 96 dots per inch. We use the resize () function from the Python programming language to retain the usual picture size of 300*30 pixels. The dimensions of the photographs are being adjusted to be the same. To rotate the picture, zoom, alter the brightness range, and perform other operations on the image, we utilise the ImageDataGenerator function from keras. preprocessing.

**Fig. 4:**
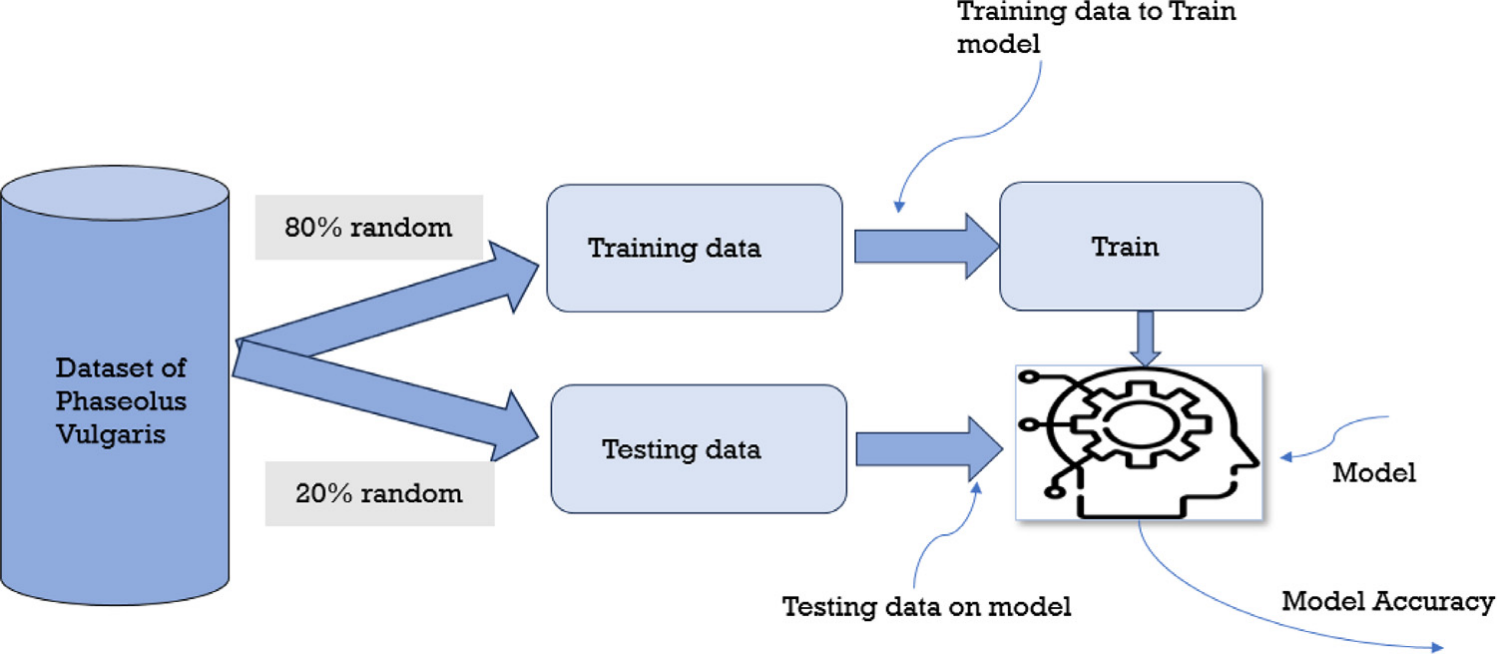
Splitting of dataset for machine learning model.

The third phases are to split the dataset for training and testing as shown in Fig. 4.

## Limitations

‘None’.

## Ethics Statement

The article “An India soyabean dataset for identification and classification of diseases using computer-vision algorithms” the following is fulfilled:

1. This article is the authors’ own original work, which has not been previously published elsewhere.

## Credit Author Statement

Jameer Gulab Kotwal: collection of dataset and preprocessing. Dr. Ramgopal Kashyap: data augmentation, methodology. Dr. Shafi Pathan: Review, writing and editing.

## Data Availability

An India soyabean leaf dataset (Original data) (https://data.mendeley.com/datasets/bshkvgbzpt/1).
